# Mosquitoes as Suitable Vectors for Alphaviruses

**DOI:** 10.3390/v10020084

**Published:** 2018-02-14

**Authors:** Elisa X. Y. Lim, Wai Suet Lee, Eugene T. Madzokere, Lara J. Herrero

**Affiliations:** Institute for Glycomics, Griffith University, Gold Coast Campus, Southport, QLD 4215, Australia; elisa.lim@griffithuni.edu.au (E.X.Y.L.); waisuet.lee@griffithuni.edu.au (W.S.L.); eugene.madzokere@griffithuni.edu.au (E.T.M.)

**Keywords:** arbovirus, vector competence, mosquito, infection, barriers, parasites, *Wolbachia*, microfilaria

## Abstract

Alphaviruses are arthropod-borne viruses and are predominantly transmitted via mosquito vectors. This vector preference by alphaviruses raises the important question of the determinants that contribute to vector competence. There are several tissue barriers of the mosquito that the virus must overcome in order to establish a productive infection. Of importance are the midgut, basal lamina and the salivary glands. Infection of the salivary glands is crucial for virus transmission during the mosquito’s subsequent bloodfeed. Other factors that may contribute to vector competence include the microflora and parasites present in the mosquito, environmental conditions, the molecular determinants of the virus to adapt to the vector, as well as the effect of co-infection with other viruses. Though mosquito innate immunity is a contributing factor to vector competence, it will not be discussed in this review. Detailed understanding of these factors will be instrumental in minimising transmission of alphaviral diseases.

## 1. Alphavirus Transmission and the Mosquito Life Cycle

The term arbovirus collectively describes viruses which are transmitted by arthropod vectors, and include alphaviruses such as chikungunya (CHIKV), o’nyong-nyong (ONNV), Ross River (RRV) and Barmah Forest (BFV) viruses. Arboviruses require a host for replication (usually birds, small mammals or non-human primates) and a vector (mainly mosquitoes) for transmission to other susceptible organisms [1]. In the arbovirus transmission cycle, the female mosquitoes acquire infection by blood feeding on a viraemic host. This virus then undergoes an extrinsic incubation period, whereby replication of the virus occurs in the mosquito midgut and disseminates to the salivary glands for transmission during the subsequent bloodmeal [2,3].

**Figure 1:**
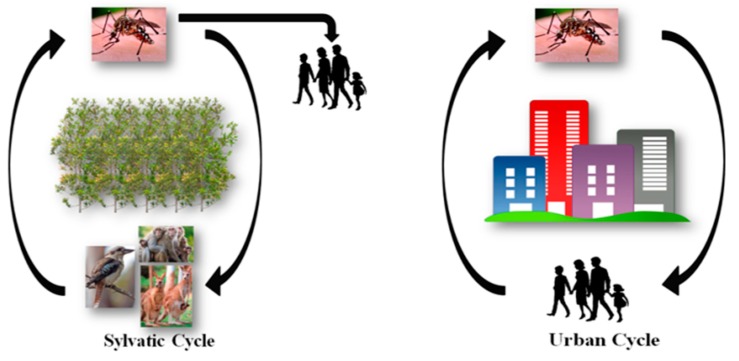
Alphaviral transmission: the sylvatic and urban cycles.

As humans are not the only hosts for arboviruses, the viruses circulate in two transmission cycles commonly known are the sylvatic cycle and urban cycle (Figure 1). For instance, the sylvatic cycle of CHIKV circulates between non-human primates and arboreal, canopy-dwelling *Aedes* species mosquitoes, and human outbreaks are uncommon [3,4,5]. On the contrary, in the urban transmission cycle, CHIKV circulate between mosquito vectors and human hosts, with humans often acting as viral reservoirs. The main mosquito vectors involved in this cycle are *Aedes aegypti* and *Aedes albopictus*, which live in close association with humans and, therefore, are responsible for medically significant human outbreaks [4]. The transmission cycle of RRV and BFV involves a cycle between kangaroos and wallabies, and includes a number of different mosquito vector species of which *Aedes vigilax* and *Culex annulirostris* are the most important [6,7,8].

The mosquito life cycle is a complete metamorphosis, comprised of four stages: egg, larvae, pupae and adult. An entire cycle will require approximately two weeks and is highly dependent on temperature and availability of water [9]. A female mosquito typically lays three to five batches of eggs throughout her lifespan of two to four weeks [10,11]. After taking a bloodmeal, the female mosquito lays her eggs on, or just above, stagnant water surfaces. To maximise the survival of her progeny, the eggs are distributed at several different sites [10]. Mosquito eggs from most genera hatch into larvae within two to three days, depending on the water temperature [11]. However, some eggs have the ability to undergo delayed hatching, where the eggs remain viable and are able to resist desiccation for up to six months. Hatching then occurs when the eggs are exposed to flooding [10]. The four larval stages take approximately one week and as the larvae progress through each stage, they molt and increase in size. After completion of the fourth larval stage, they transition to a non-feeding pupal stage. When development is complete (this takes approximately two days), the mosquito emerges from the pupal case [10].

## 2. Factors of Vector Competence

The permissiveness of a vector to acquire an infection, undergo replication and transmit a virus is known as vector competence [12,13]. Not all haematophagous mosquitoes are vectors of arboviruses, and there are many factors implicated in determining the competency of a vector. Other extrinsic factors are environmental temperature, rainfall and mosquito fitness [14,15].

**Figure 2:**
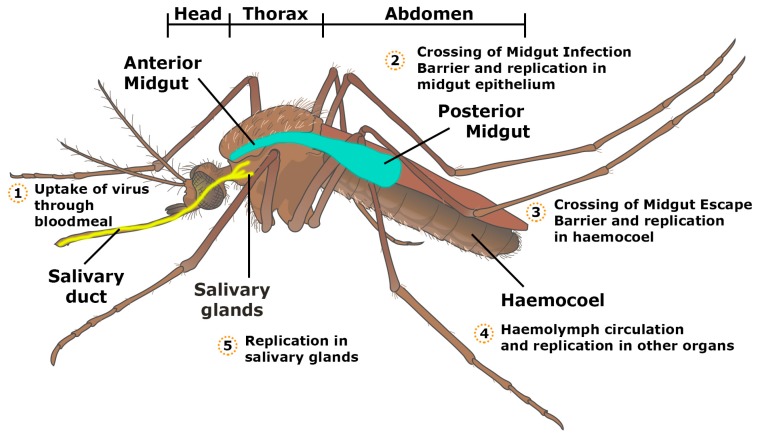
Schematic representation of the locations of virus replication in the mosquito.

In the model for a productive virus infection in mosquitoes, proposed by Hardy et al., the virus in the ingested bloodmeal has to infect and replicate in the epithelial cells of the midgut. The virus must then successfully escape from the midgut and infect the salivary glands and ovaries, followed by release of the virus into the salivary ducts for transmission orally to vertebrates or transovarially to the mosquito’s offspring (Figure 2) [15]. Salivary gland infection and escape barriers determine if the virus can replicate and shed into the mosquito’s saliva for final transmission to the vertebrate host during a bloodmeal [16,17]. Factors affecting the ability of the virus to pass through these barriers remain largely unknown, however, some studies have suggested that infecting viral dose affects the rate of dissemination of the virus through these barriers, e.g., mosquitoes with high virus titre transmit the virus more effectively [16,17].

The mosquito’s potential to cause disease can be estimated via mathematical models of vectorial capacity [18,19,20]. The classical model, also the most influential, is the Ross-MacDonald mathematical model of pathogen transmission via mosquitoes. It measures the number of infectious bites that could potentially occur from all the mosquitoes that could bite an infectious person in a given day [18,19]. The populations, compositions and behaviours of both mosquito and vertebrate hosts influence the chances of vector contact with susceptible vertebrate hosts [14,15]. As such, parameters such as vector population density, longevity, blood-feeding rates and vector competence are taken into consideration when assessing vectorial capacity.

Though vectorial capacity and vector competence are both terms used to describe the vector’s potential to cause disease, the ability of the mosquito to become infected and successfully transmit the virus following ingestion of a bloodmeal is known as vector efficiency or vector competence [12,13,15]. This review focuses on factors that influence vector competence such as the physical tissue barriers which the virus has to overcome, genetic variability between virus strains and the effect of mosquito microenvironment and external environmental variables on virus infection and transmission.

## 3. Tissue Barriers to Infection in the Mosquito Vector

**Figure 3:**
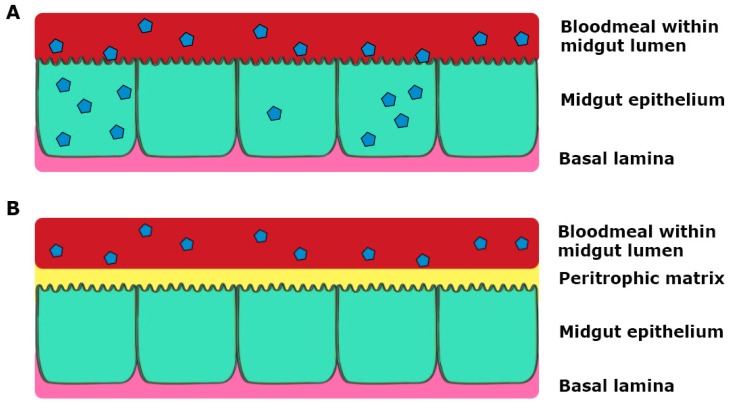
Schematic representation of infection of the midgut, where virus particles are represented as blue polygons. (**A**) Virus in the bloodmeal infects epithelial cells through the microvilli and replicates; (**B**) Virus in the bloodmeal fails to infect the epithelial cells prior to secretion of the peritrophic matrix by midgut epithelial cells during blood digestion.

After a bloodmeal from an infectious individual, the ingested virus must be able to replicate in the mosquito midgut epithelium, and subsequently the salivary glands, in order for successive infection to a vertebrate host (Figure 2) [12,21]. The virus from the bloodmeal penetrates into the midgut epithelium by crossing the first cellular barrier, the midgut infection barrier (MIB) [22]. The midgut and its associated tissue barriers are one of the first obstacles the virus has to overcome for successful viral replication and transmission. The midgut comprises of a layer of epithelial cells, which contains the bloodmeal post-feeding. Following bloodfeeding, the midgut epithelial cells secrete peritrophic matrix into the lumen during the blood digestion process, which envelops the bloodmeal and prevents virions from gaining access to the epithelial cells (Figure 3). Therefore, the virus has to infect the epithelial cells prior to secretion of the peritrophic matrix or passing through the peritrophic matrix [23,24].

Once in the midgut epithelium, the virus replicates and escapes into the haemocoel, by crossing the midgut escape barrier (MEB) into the basal lamina [12,21,25,26]. Recently, it has been shown that the basal lamina could be a potential tissue barrier to alphavirus infection [23]. Dong et al. compared virus dissemination in sugar-fed and bloodmeal-fed *Aedes aegypti* infected with CHIKV intrathoracially. Using immunofluorescence and electron microscopy techniques, they observed that the virions were associated with the basal lamina in sugar-fed mosquitoes and were unable to infect the midgut epithelium [23]. In mosquitoes fed through bloodmeal, CHIKV virions were able to cross the basal lamina and infect the midgut epithelial cells [23].

Once the virus crosses the MEB, it enters the haemocoel, an open body cavity in which haemolymph circulates. Haemocytes are the immune cells present in the mosquito’s open circulatory system and together with haemolymph circulation, are crucial components for viral dissemination [27]. Within the haemocoel, the virus is able to infect other secondary tissues, such as the muscle, trachea, fat bodies and salivary glands [28,29,30].

Salivary glands are a crucial and final physical barrier that is important for effective transmission of arboviruses. The ability of the virus to traverse to the salivary glands varies depending on the virus strain and mosquito species. The salivary gland of *Anopheles stephensi* is surrounded by basal lamina that prevent binding of virus to cell surface receptors, thus acting as a barrier to prevent infection [31]. The time required from the acquisition of virus to replication, dissemination and transmission through saliva is reliant on the ability of the virus to infect the salivary glands. For example, CHIKV could be detected in the saliva of infected *Aedes aegypti* as early as four days post-infection [32]. Sindbis virus (SINV) infection results in morphological changes and pathology in the salivary gland of *Aedes albopictus* [33]. When a sufficient amount of the virus is present, the mosquito is able to transmit the virus during feeding, by injecting infected saliva into a vertebrate host. Mosquito saliva contains compounds that enhance vasodilation and prevent blood coagulation in the vertebrate host [34,35,36]. Furthermore, during mosquito bites, virus infection and spread is enhanced through the recruitment of neutrophils and myeloid cells as part of the inflammatory response [35]. Mosquito bites also result in edema which aids the retention of virus in the skin, thus allowing for the infection of cutaneous cells [35]. For example, subcutaneous infection of Semliki Forest virus (SFV) in mice in the absence of mosquito bites resulted in drainage of the virus three to six hours post-infection and this is hypothesised to be the cause of the low viraemia found and poor spread to remote tissues [35]. The extrinsic incubation period (EIP) is the time needed between acquisition of the virus and the point at which the mosquito is able to infect a new vertebrate host during a subsequent bloodmeal [26,37]. The EIP varies among virus genotype and mosquito strain, and is greatly dependent on environmental factors, such as temperature and effective dose of virus [16,30]. The EIP is seven to ten days for CHIKV [29] and virions can be detected in the salivary glands of *Aedes aegypti* as soon as two days after oral exposure to the virus. However, a minimum of one week is required for virus to be released and detected in saliva, indicating absence of salivary gland escape barrier and presence of salivary infection barrier [38].

## 4. The Roles of Parasites in Alphavirus Infection

Mosquitoes are frequently infected with parasites that may play a role in alphavirus-mosquito interactions [39]. Microfilariae are an early developmental stage of certain parasites which are known to live in vertebrates. As part of their life cycle, the adult parasites release microfilariae into the host’s bloodstream and these microfilariae become ingested by mosquitoes, where they mature into infectious larvae. Microfilariae are known to penetrate the midgut within minutes of ingestion by the mosquito and migrate to the haemocoel. In doing so, virions present in the bloodmeal are able to enter the midgut and haemocoel through the entry and exit holes created by microfilariae as it passes through the epithelial barrier. This facilitation of viruses across the midgut barrier is known as microfilarial enhancement of arboviral transmission. It is hypothesised that this results in greater vector competence, as less susceptible mosquitoes are infected by viruses, increasing the number of infected mosquito vectors during a transmission cycle [39]. Additionally, the shorter virus incubation period allows the virus to be transmitted by the mosquitoes quickly, leading to enhanced arboviral transmission [39].

Although microfilarial enhancement appears to be dose-dependent, the levels of enhancement also varies among mosquito species [40]. In a study by Vaughan and colleagues, three species of *Aedes* mosquitoes were allowed to feed on gerbils co-infected with *Brugia malayi* microfilaria and Eastern equine encephalistis virus (EEEV). The authors found decreasing levels of microfilarial enhancement of EEEV in *Aedes taeniorhynchus*, *Aedes aegypti* and *Aedes triseriatus* respectively and hypothesised that this may be due to differences in the amount of blood leaked into the hemocoel upon microfilarial penetration [40]. Consequently, a follow-up study was performed using Venezuelan equine encephalitis virus (VEEV) [41]. Despite the lower doses of VEEV and *Brugia malayi* microfilaria used, the level of microfilarial enhancement was greater compared to the earlier study with EEEV [41]. The mosquitoes were also less susceptible to VEEV, suggesting that factors apart from bloodmeal leakage play roles in microfilarial enhancement of arboviral transmission [41].

In a recent study done by the same group, it was found that EEEV and VEEV binds to *Brugia malayi* microfilaria, allowing it to be transported across the midgut epithelial barrier and suggesting this as the main mechanism for microfilarial enhancement [39]. Comparing co-infection of virus and microfilaria with direct virus inoculation into the haemocoel, the authors found a delay of 24 h in the growth kinetics of co-infected virus in the haemocoel, and postulates that this delay is due to the time required for microfilaria penetration, as well as the amount of virus delivered [39]. Another set of experiments were performed using multiple vigorous washings of microfilaria after virus incubation and infectious virus was still detected, suggesting viral attachment to the microfilaria [39].

Another example of parasites that play roles in alphavirus infection is the *Wolbachia* bacteria which has been extensively studied for the potential use in mosquito and virus transmission control. This bacteria is naturally present in several mosquito species which are vectors of human pathogenic viruses such as *Aedes albopictus*, also known for its wide geographical distribution [42]. Interestingly, *Aedes aegypti* has not been found to be naturally infected with *Wolbachia* [42,43,44]. Nevertheless, studies have demonstrated that *Aedes aegypti* can be infected with *Wolbachia*, despite the lack of evidence of natural infection [43,45]. *Aedes aegypti* infected with the *w*Mel strain of *Wolbachia* have been shown to have reduced vector competence for CHIKV [45]. The authors found no infectious virus in the saliva within the seven days post feeding period [45]. They also demonstrated that *w*Mel *Wolbachia* infection did not affect the lifespan of the mosquitoes, suggesting that this is a possible means of minimising CHIKV transmission [45]. A similar study conducted using *Aedes aegypti* from Singapore found reduced infections of CHIKV in *w*Mel-carrying mosquitoes as compared to wild-type mosquitoes [43]. Though some of the *w*Mel-carrying mosquitoes in this study were found to have CHIKV replicating in their midguts, the number of mosquitoes positive for CHIKV in their salivary glands was minimal, suggesting the role of *w*Mel in preventing CHIKV dissemination to the salivary glands and blocking virus transmission [43]. Another recent study found a high infection rate of natural *Wolbachia* clades *w*AlbA and *w*AlbB in *Aedes albopictus* from several different geographical regions in Malaysia [42]. Upon further characterisation of these naturally infected mosquitoes, Ahmad et al. found that *Wolbachia* colonisation did not inhibit CHIKV replication, and hypothesised that this may perhaps explain how this mosquito strain is able to serve as a competent vector for CHIKV [42]. However, it is difficult to determine if genetically engineered strains of *Wolbachia,* which have lower vector competence, could displace the existing *Wolbachia* populations found in the mosquitoes [42].

Despite the abundance of *Wolbachia*-related literature published, the mechanisms of how *Wolbachia* colonisation in mosquitoes could result in reduced vector competence for viruses remains poorly understood. *Wolbachia* density in mosquitoes may possibly be correlated to its antiviral properties. Non-native *Wolbachia* strains have also been shown to inhibit virus infection better compared to native *Wolbachia* strains. An example of this is demonstrated in a recent study by Schultz et al., whereby the *w*Stri strain (native to the leafhopper *Laodelphax striatella*) was more effective than *w*AlbB (native to *Aedes albopictus*) in Zika virus (ZIKV) inhibition in C710 (*Aedes albopictus* cell line) [46]. Indeed, many mosquito or cell culture based studies involve the use of non-native *Wolbachia* strains [43,44,45,46,47,48]. Coincidentally, *w*AlbB grows to higher density per cell in *Aedes aegypti* compared to *Aedes albopictus*, its native host [49]. The phenomenon known as the density-dependent phenotype could possibly explain the lack of CHIKV inhibition in naturally *w*AlbA- or *w*AlbB-infected *Aedes albopictus* mosquitoes in the field study by Ahmad et al. [42]. However, Amuzu et al. characterised the *Wolbachia* densities and dengue virus (DENV) loads present in various tissues of *Wolbachia*-infected and uninfected mosquitoes but were unable to find any correlation between *Wolbachia* densities and DENV inhibition. The authors also found that DENV inhibition by *Wolbachia* was greatest at early stages of virus infection. This is consistent with findings by Rainey et al., who investigated the molecular aspects of *Wolbachia*-based Semliki SFV inhibition in Jw18 (*Drosophila melanogaster* cell line), and found that virus replication was inhibited as early as 7 h post infection [47]. The antiviral effects modulated by *Wolbachia* were found to be constitutively active as the authors were unable to detect any significant changes in *Wolbachia* gene expression upon virus infection [47]. There were also no differences found in host miRNA response during SFV infection in the presence and absence of *Wolbachia*, suggesting that the antiviral effects observed were attributed to *Wolbachia* and not mediated by the host’s antiviral response [47]. Other mechanisms of pathogen blocking by *Wolbachia* such as host immune priming and competition for host resources are discussed in a recent review by Terradas and McGraw [50]. Additionally, virus inhibitory effects caused by Wolbachia does not appear to be affected by the gut bacteria flora [51]. This is consistent with data from another study in which Wolbachia appears to play a dominant role in gut microflora in *Aedes albopicus* and *Culex pipiens* mosquitoes [52].

Though we have thus far introduced microfilariae and *Wolbachia* bacteria, there are other key players in the mosquito gut microflora that influence virus infection as well. Of interest is a recent and comprehensive report by Angleró-Rodríguez et al., which describes the first fungus that is found naturally associated with the mosquito gut and is involved in virus infection [53]. The *Talaromyces* sp. fungus (designated *Tsp_PR*) was found in the midgut of field *Aedes* sp. mosquitoes and its identity confirmed through sequence analysis [53]. The introduction of either *Tsp_PR* spores or secretome resulted in increased DENV replication in the midgut [53]. However, when the secretome was heat-treated, the enhanced DENV infection effect was not observed, indicating that the components responsible for DENV enhancement could be heat-sensitive [53]. Interestingly, when *Tsp_PR* was replaced with another fungus strain previously isolated from field mosquitoes, *Penicillium chrysogenum*, no effect on DENV infection was found [53]. The presence of *Tsp_PR* also did not have any effect on the bacteria flora present in the midgut, neither did it affect mosquito longevity [53]. The authors further investigated the effect of *Tsp_PR* secretome on mosquito transcriptional activity and found that many down-regulated genes were involved in bloodmeal digestion, which may explain the enhanced DENV infection observed [53].

Many studies focus on characterising the effect of *Wolbachia* to virus infection and often, the possible roles of the other bacteria present in the mosquito gut microflora is neglected. An example of another bacteria which displays anti-pathogenic properties is *Csp_P*, a *Chromobacterium* sp. isolated from field-caught *Aedes aegypti* mosquitoes [54]. This bacterial strain colonises the midguts of *Anopheles gambiae* as well [54]. It is able to inhibit malaria parasite *Plasmodium falciparum* and DENV infection in mosquitoes and it secretes molecules with anti-pathogenic activity [54]. However, this bacteria is not suitable for use in vector control as exposure to it results in reduced lifespan of larvae and adult mosquitoes [54].

## 5. Mechanisms of Viral Adaption to Vector

### 5.1. Effect of Different Viral Strains (or Mutations) on Vector Competency

Alphaviruses can be genetically diverse, which contribute to phenotypic plasticity and adaptability to dynamic environments, including new host species [55]. For example, RRV infects over 20 vertebrate hosts and has been isolated from over 30 mosquito species [56]. As RNA viruses, alphaviruses have RNA-dependent RNA polymerase (RdRp) encoded into their genome. This enzyme is responsible for viral genome replication and has a high error rate that can generate one mutation for every 10 kilobases copied [57]. Therefore, this results in higher mutation rates and contributes to genetic diversity. Single nucleotide mutations of N-linked glycosylation sites on viral glycoproteins dramatically impact both the transmission and survival of arboviruses, as well as mosquito vector competence [58].

Alphaviruses are also able to effectively employ a motif mimicry-based strategy to productively infect and adapt to both their target hosts and vectors. Here, hijacking of host and/or vector cellular and functional processes through viral motif mimicry is modulated by small, linear and functionally constrained poly-amino acid motifs (approximately 3 to 10 amino acid residues in length) called Short Linear Motifs (SLiMs) [59]. While short in length, these viral SLiMs contain at least five degenerate positions; they are evolutionarily plastic and particularly amenable to point mutations and convergent evolution, and they are usually housed within disordered regions in structural and non-structural genes [59,60,61,62,63].

The Indian Ocean island CHIKV epidemics of 2005–2006 provided the best example of how point mutations and/or convergent evolution within alphaviral SLiMs can influence viral adaptation to a specific mosquito vector [64]. This CHIKV outbreak was initiated by a strain expressing an E1 envelope glycoprotein with the amino acid alanine (A) at position 226 (A226) [65]. However, as the epidemic progressed, CHIKV strains isolated from the same geographic region expressed either the alanine (A226) or valine residues at position 226 (V226) in the same E1 glycoprotein, and eventually the new V226 genotype largely dominated in infected humans [65].

Extensive analyses revealed that this mutation (E1-A226V) was directly responsible for an increase in CHIKV infectivity for *Aedes albopictus*, thus improving viral dissemination and the transmission to suckling mice experimentally without affecting viral fitness in *Aedes aegypti* [66,67]. Quite remarkably, this mutation was acquired independently in several distinct geographical locations (India and West Africa) where, similar to La Reunion, *Aedes albopictus* is widely present and is actively displacing indigenous *Aedes aegypti* populations. This suggests that by altering the vector specificity or preference for CHIKV strains, the (E1-A226V) mutation also influenced both the vectorial capacity and vector competence of the *Aedes albopictus* population resulting in CHIKV strains carrying this mutation achieving what is widely recognized today as a unique spatial global distribution. Apart from the discovery of a point mutation or a convergent evolutionary event that occurred at position 226 of the E1 glycoprotein, very little is known regarding the precise molecular mechanisms by which CHIKV fitness (infection, dissemination and transmission) was increased in *Aedes albopictus*. The identities of the SLiM residues flanking this E1 envelope glycoprotein mutational site (226) are also unknown. 

Follow-up studies initially suggested that the E1-A226V mutation caused an increase in the dependency on cholesterol during the virus-host cell fusion [66,68]. This postulation was based on the observation that mutated CHIKV isolates displayed an attenuated viral growth in C6/36 mosquito cells devoid of cholesterol, as compared to original, non-mutated strains [66,68]. This differential phenotype was suspected to be an additional factor for the preferential replication in *Aedes albopictus*. It has been shown that the E1-226V residue is located at regions of fusion with host cell membranes [69]. Furthermore, in *Aedes albopictus,* the E1-226V variant has a greater capacity to cross the midgut barrier and infect local cells compared to the E1-226A variant [70]. Additionally, the E1-226V variant loses cholesterol dependence for growth [71]. Further investigations revealed that there is no clear correlation between the dependence on cholesterol and the capacity of CHIKV to infect *Aedes albopictus*, suggesting that these are two independent phenotypic effects of the E1-226 mutation [72].

The evolutionary constraints imposed by epistatic interactions between residues 226 and 98 of the E1 glycoprotein may be the reason why the E1-A226V mutation only appears in East-South-Central African (ECSA) CHIKV strains and not in the Asian CHIKV strains circulating in areas where *Aedes albopictus* is common [73]. Certainly, all endemic Asian CHIKV strains have been observed to contain a threonine in position 98 that is absent in both Indian Ocean Island (IOL) and ECSA CHIKV strains and consequently this genotype limits the adaptive effect of the E1-A226V mutation in *Aedes albopictus* [73]. There is a great possibility that due to this constraint, the ongoing American CHIKV epidemics, caused by Asian strains, may be sustained by *Aedes aegypti* instead of *Aedes albopictus* in areas where they are sympatric [74]. However, this dynamic may change if the introduced *Aedes albopictus-*fitted ECSA and IOL strains settle in the area [75].

“Second-step” adaptive mutations have also been described in E1-A226V CHIKV strains that further potentiates viral replication in *Aedes albopictus* [76]. The most notable of these second-step adaptive mutations consists of a leucine for glutamine substitution in position 210 (L210Q) of the E2 envelope glycoprotein, which facilitates viral binding. This E2-L210Q mutation, characterised in viral isolates from Kerala in Southwest India [76], facilitates infection of midgut epithelial cells, thereby increasing viral dissemination and transmission by *Aedes albopictus* [77]. The E2-L210Q mutation does, however, have a less significant effect on *Aedes aegypti* as compared to its effect on *Aedes albopictus* [77]. In addition, an experimental study conducted by Stapleford et al. showed the emergence of two new mutations V80I and 129V on E1 glycoprotein of the CHIKV A226V strain [78]. Positive selection of these mutations appears to improve the stability and fusogenic activity of these variants in *Aedes albopictus* [78]. These lines of evidence clearly indicate that some CHIKV strains are rapidly evolving to exploit *Aedes albopictus* as a major vector in areas where it is abundant. For instance, in Europe and North America where *Aedes albopictus* mosquitoes are rapidly spreading, there is heightened concern about the epidemic potential of mutant CHIKV strains [75].

Several studies have also reported increased competence for CHIKV as compared to DENV in both *Aedes albopictus* and *Aedes aegypti* but with no indication of the underlying molecular determinants driving increased competence. In a French study, researchers showed that *Aedes albopictus* was more competent for CHIKV compared to the DENV-2 virus [79]. Furthermore, CHIKV could be transmitted from the mosquito as early as two days after ingestion of infected blood by the mosquito, with 1000 viral RNA molecules being detected in the salivary glands [79]. A similar study was conducted in Thailand where the researchers concluded that the rate of multiplication and oral receptivity of CHIKV was faster in laboratory-bred *Aedes aegypti* as compared to DENV [80]. Whether CHIKV strains used in the aforementioned French and Thailand vector competence studies contained either the E1A226V, E2L210Q, E1V80I, E1129V or other new mutations is unknown. In Australia, the mutations, convergent evolutionary events and/or viral SLiMs modulating increased competence in *Aedes vigilax* (Skuse) and *Aedes notoscriptus* mosquito populations [81,82,83], for both BFV and RRV, have not been elucidated.

By contrast, the molecular determinants that drive distinct mosquito vector adaptations in ONNV were recently located within the non-structural protein 3 (nsP3) region of the virus [84]. The nsP3 region of the alphavirus consists of a macro or amino-terminal domain that is highly conserved and a carboxy-terminal domain that is highly variable in size and sequence and devoid of any secondary structure [85,86]. The nsP3 region has also been implicated in the correct formation and localization of replication complexes, and provision of essential functions to both minus strand and subgenomic RNA synthesis [84]. Moreover, it was demonstrated that when ONNV nsP3 replaced the nsP3 from CHIKV in chimeric viruses, infection rates in *Anopheles gambiae* rose from 0 to 63.5% [84]. These authors further postulated that dividing the two nsP3 domains may serve to disrupt alphavirus-SLiM-vector interactions or remove SLiMs or residues constituting functionally relevant SLiMs required for successful vector capacity and vector competence. Other studies have detected several foreign genetic elements inserted through mutations in the carboxy-terminus domain of the alphavirus nsP3 region. In one such study, an eight-amino-acid SLiM found in the carboxy-terminus of CHIKV nsP3 was mapped to the putative zinc-finger protein in *Aedes aegypti*, the main vector of the virus [87]. How this CHIKV SLiM influences the vector capacity and competence in *A. aegypti* remains unknown. However, studies with the alphavirus SINV, have shown that deletions in the carboxy-terminus rendered mutants defective in initiating a productive infection, generating plaques in mosquito cells at only 1–2% the efficiency of the parental virus [88]. Although a single mutation in this nsP3 carboxy-terminus proline-rich SLiM in SFV or SINV is reported to have greatly impaired RNA synthesis by disrupting the host amphiphysins, the effect of the same mutation on vector competence has yet to be determined [89]. It is currently suspected that the nsP3 carboxy-terminus proline-rich SLiM (PIPPPR) shared by many alphaviruses, modulates ONNV vector competence since ONNV and CHIKV differs by only one amino acid substitution in the residue pattern of this particular SLiM. Investigations into alphaviral SLiM-vector interactions, particularly those influencing adaptation to the vector, vector competence and capacity, must be encouraged and supported as to minimize the spread of alphaviruses globally.

### 5.2. Effect of Temperature on Alphaviral Vector Competence

Predicting the effects of climatic variables, such as temperature, on vector competence for alphaviruses has been challenging. Very little is currently known regarding how alphavirus-vector interactions are modulated. In particular, the role of climatic variables such as temperature. Temperature is an important biotic variable that directly affects the mosquito biting rate, fecundity (egg per female per day), egg to adult survival, mosquito development rate, adult lifespan, the extrinsic incubation rate, and infection and transmission probabilities [90]. Higher temperatures have conflicting effects on mosquito competence for pathogenic viruses; higher temperatures can either shorten the time it takes mosquitoes to become competent vectors of human viral pathogens [91,92], or they can shorten the lifespan of successful mosquito vectors [93]. An inverse relationship appears to exist between the temperature at which mosquito larvae undergo development and their future susceptibility to alphavirus infection [94,95]. In particular, lower temperatures have been shown to adversely affect a vector’s ability to modulate viral infection and increases the rates of transovarial transmission [96,97,98,99]. Kramer et al. demonstrated that infection levels of Western equine encephalitis virus (WEEV) in *Culex tarsalis* decreased as a function of increasing temperature [97]. This study hypothesized that mosquitoes were better able to modulate virus infection at higher temperatures. This ability to modulate virus infection was inheritable, suggesting a genetic basis for the phenotype [97]. Similar findings were made by Kay and Jennings using RRV in *Aedes vigilax* kept at 18 °C [96]. Another study by Turell found significantly higher rates of disseminated infections for both Rift Valley Fever virus and VEEV following the ingestion of an infectious bloodmeal when mosquitoes were kept at 19 °C as compared to 26 °C [95]. Westbrook et al. tested several rearing temperatures (18 °C, 24 °C, 32 °C) and also found that the infectivity of CHIKV for *Aedes albopictus* increased at lower rearing temperatures [94].

The environmental temperature may affect mosquito physiology, thereby affecting viral replication and transmission. Muturi et al. evaluated the effect of *Aedes aegypti* larval rearing temperature and intraspecific larval competition on vector competence for SINV and found a direct relationship between *Aedes aegypti* larval density and SINV infection and dissemination rates at low temperature (20 °C) and an inverse relationship between larval density and SINV infection rate at high temperature (30 °C) [100]. In another study done in New Zealand, *Aedes antipodeus*, was shown to be efficiently infected with BFV and RRV at low extrinsic incubation temperatures simulating winter conditions (16 °C) where the mosquito vector was able to transmit each of the viruses after 21 days or earlier [101]. It has also been suggested that *Aedes antipodeus* mosquito vector populations should be monitored as they are potentially competent vectors for RRV and BFV transmission in New Zealand [101]. In the United States of America, epidemics of WEEV prevail above a 21 °C isotherm and the primary vectors of WEEV are *Culex tarsalis Coquillett* mosquitoes. Furthermore, Reeves et al. evaluated the effect of temperature changes on the survival of this mosquito vector species and revealed that, (i) daily mortality of adult vectors increased by 1% for each 1 °C increase in temperature; (ii) only 5% of *Culex tarsalis* survived for eight or more days at 25 °C, the time required for extrinsic incubation of WEEV; (iii) increasing temperatures from 18 to 25 °C resulted in shortened extrinsic incubation times for WEEV, and that; (iv) WEEV infection and transmission decreased at 32 °C [102]. This demonstrates the effect of temperature on mosquito populations and alphaviral transmission.

### 5.3. Co-Infection with Other Viruses

One of the most likely outcomes of viral co-infection is a phenomenon known as superinfection exclusion, where primary infection with a specific viral strain prevents secondary infections with either the same or other genetically distinct viral strains. Karpf et al. reported that three *Aedes albopictus* cell lines (e.g., U4.4, C6/36 and C7-10) persistently infected with the HR strain of SINV were able to prevent replication of both homologous (various strains of SINV) and heterologous alphaviruses (such as Aura virus, SFV and RRV) [103]. Though these SINV-infected cells were able to exclude infection by other alphaviruses, they were susceptible to infection by Yellow fever virus, a known flavivirus. These authors also hypothesised that superinfection exclusion is conserved amongst *Aedes albopictus* cell lines [103]. Broadly, conflicting results have been obtained regarding the ability of alphaviruses to exclude other species of alphaviruses in persistently-infected mosquito cells. For example, an uncloned line of *Aedes albopictus* cells persistently-infected with SINV failed to replicate superinfecting SINV [104]. However, when a different species of alphavirus EEEV was used for the superinfection, the virus titre detected was reported to be similar to that in EEEV infection in normal cultures [104]. A follow-up investigation by Eaton later showed that SINV-infected *Aedes albopictus* cells efficiently excluded several heterologous alphaviruses (CHIKV, Una, and SFV) if the cells were superinfected at early times after primary SINV infection, but excluded them much less efficiently if superinfection occurred after long-term persistent infection [105]. The interpretation of these two results was complicated by the use of different alphaviruses, and by the use of uncloned cell lines, as the original cell line derived from mosquito larvae by Singh is now known to contain a number of distinct cell types which have variable responses to alphavirus infection [106,107,108,109]. These cellular distinctions include the appearance of cytopathology during acute phase infection [107,108,109], the route of viral maturation, and the distribution of the viral proteins within the infected cell types [107,108,109].

Virus co-infection could also occur between virus families and several studies have presented the impact of alphavirus and flavivirus co-infection on the vector competence of *Aedes* genus mosquitoes [110,111]. For example, Rückert et al. recently evaluated the impact of co-infection on the ability of *Aedes aegypti* to transmit CHIKV, DENV-2 or ZIKV, individually and as double and triple co-infections [110]. They found that *Aedes aegypti* was susceptible to infection for all the combinations tested and was also able to transmit the viruses simultaneously. Moreover, the rates of infection, dissemination and transmission were not significantly affected by co-infection of these viruses [110]. In a related study, *Aedes albopictus* was shown to be amenable to oral co-infection with both DENV and CHIKV E1-226V [112]. Herein, DENV and CHIKV were able to replicate simultaneously in *Aedes albopictus* and this vector was noted to retain the ability to deliver infectious particles of both viruses in a single bite via saliva [112]. Furthermore, the same study also demonstrated that a secondary CHIKV infection could be introduced in *Aedes albopictus* carrying a DENV primary infection. The identification of SLiMs may be helpful in elucidating the mechanisms behind superinfection. For instance, the tripeptide Arginine-Glycine-Asparagine RGD motif is a SLiM found in the envelope proteins of alphaviruses and flaviviruses [113]. This SLiM is involved in the binding of virus particles to human host integrins and may facilitate virus entry into cells [113]. This suggests that alphaviruses and flaviviruses could possibly encode for and share similar viral SLiMs which are able to potentiate interactions with cell surface receptors on mosquito vectors thereby allowing for superinfection. Since analyses of CHIKV proteins by Mathur et al. recently revealed that non-structural proteins nsP2 and nsP3 exhibit RNA interference (RNAi) suppressor activity, it is possible that the same or other non-structural proteins may harbour superinfection exclusion suppressor activity [114]. Additionally, another recent study has shown simultaneous infection, dissemination and transmission of DENV and CHIKV in two mosquito species, *Aedes aegypti* and *Aedes albopictus* [115]. In this study, groups of mosquitoes were orally infected with DENV and CHIKV simultaneously or sequentially. Mosquitoes were then tested for their potential to disseminate and transmit both viruses simultaneously by quantitative RT-PCR. Simultaneous dissemination of DENV and CHIKV was detected in both species of mosquitoes [115]. The authors also observed a lower rate of dissemination of both viruses when administered simultaneously as compared to the sequential infection in which a significantly higher rate of dissemination of both the viruses was found [115]. In contrast to the results obtained by Vazeille et al., an in vitro study conducted using the *Aedes albopictus* C6/36 cell line characterized the co-infection dynamics of DENV-3 and CHIKV (ECSA genotype) and concluded that; (i) higher titres of DENV-3 resulted in competitive suppression of the replication of CHIKV and; (ii) DENV-3 and CHIKV replications depend on virus titre rather than on serial infection [116]. There is a genuine need to conduct further investigation into the dynamics of mosquito co-infection with alphavirus and flavivirus pathogen populations considering that millions of disease cases caused by these viruses are reported globally. Furthermore, it is not uncommon to find these viruses co-circulating in certain regions. Additionally, there are currently no anti-viral treatments or vaccines available to minimize the pain and suffering endured by those affected.

## 6. Conclusions

Many alphaviruses are medically important, and emerging global pathogens due to the presence of mosquito vectors and human populations. Though different regions are hosts to different mosquito vectors, it has been shown that alphaviruses are capable of successfully adapting to their chosen vectors. Additionally, many factors that contribute to vector competence come into play, resulting in increased vector competence of mosquito vectors previously less susceptible to virus infection. There are many challenges in the control of mosquito populations and though *Wolbachia* infection is a potential vector control measure, different genetic strains may be required for different regions due to the variable mosquito populations in various geographical regions. Moreover, the *Wolbachia* strain must be compatible with the existing commensal microflora present in the mosquito midgut, and should not have any detrimental effects on mosquito longevity. Additionally, the constant evolution of the virus, *Wolbachia*, and mosquito vector makes it difficult to determine how long the effects of *Wolbachia* population displacement will last. Other targets for vector control will have to be determined and hence, further understanding of the complex interactions that affect vector competence is required.
